# Sound-Evoked Activity Influences Myelination of Brainstem Axons in the Trapezoid Body

**DOI:** 10.1523/JNEUROSCI.3728-16.2017

**Published:** 2017-08-23

**Authors:** James L. Sinclair, Matthew J. Fischl, Olga Alexandrova, Martin Heβ, Benedikt Grothe, Christian Leibold, Conny Kopp-Scheinpflug

**Affiliations:** Division of Neurobiology, Department Biology II, Ludwig-Maximilians-University Munich, 82152 Planegg-Martinsried, Germany

**Keywords:** ABR, auditory brainstem, axonal conduction speed, calyx of Held, MNTB, myelination

## Abstract

Plasticity of myelination represents a mechanism to tune the flow of information by balancing functional requirements with metabolic and spatial constraints. The auditory system is heavily myelinated and operates at the upper limits of action potential generation frequency and speed observed in the mammalian CNS. This study aimed to characterize the development of myelin within the trapezoid body, a central auditory fiber tract, and determine the influence sensory experience has on this process in mice of both sexes. We find that *in vitro* conduction speed doubles following hearing onset and the ability to support high-frequency firing increases concurrently. Also in this time, the diameter of trapezoid body axons and the thickness of myelin double, reaching mature-like thickness between 25 and 35 d of age. Earplugs were used to induce ∼50 dB elevation in auditory thresholds. If introduced at hearing onset, trapezoid body fibers developed thinner axons and myelin than age-matched controls. If plugged during adulthood, the thickest trapezoid body fibers also showed a decrease in myelin. These data demonstrate the need for sensory activity in both development and maintenance of myelin and have important implications in the study of myelin plasticity and how this could relate to sensorineural hearing loss following peripheral impairment.

**SIGNIFICANCE STATEMENT** The auditory system has many mechanisms to maximize the dynamic range of its afferent fibers, which operate at the physiological limit of action potential generation, precision, and speed. In this study we demonstrate for the first time that changes in peripheral activity modifies the thickness of myelin in sensory neurons, not only in development but also in mature animals. The current study suggests that changes in CNS myelination occur as a downstream mechanism following peripheral deficit. Given the required submillisecond temporal precision for binaural auditory processing, reduced myelination might augment sensorineural hearing impairment.

## Introduction

Transmission of signals through the nervous system is determined by the number of action potentials (APs), their temporal precision, and the speed of propagation. Some classes of auditory brainstem neurons, for example, the globular bushy cells (GBCs) of the ventral cochlear nucleus (VCN), are capable of generating instantaneous rates in excess of 1000 Hz (Taberner and Liberman, 2005; Kopp-Scheinpflug et al., 2008a,b; Sonntag et al., 2009; Typlt et al., 2012). GBCs provide excitatory input to the principal neurons of the medial nucleus of the trapezoid body (MNTB) via a giant axosomatic synapse known as the calyx of Held (Held, 1893). Calyx synapses and MNTB principal neurons express a specialized complement of voltage and ligand-gated ion channels to allow AP generation at very high frequencies (Johnston et al., 2010; Kopp-Scheinpflug et al., 2011). GBC axons constitute part of the trapezoid body (TB), a large decussating fiber tract. TB fibers support high firing rates and their diameter and myelination allow adjustment of conduction speed to ensure that binaural auditory inputs coincide at superior olivary structures. For example, GBC axons in gerbils are uniquely tuned according to their tonotopic termination site (Ford et al., 2015).

Neuronal activity enhances myelination (Demerens et al., 1996). Activity-dependent increases in the thickness and abundance of myelin have been observed in several regions of the vertebrate nervous system (Bengtsson et al., 2005; Liu et al., 2012; Schlegel et al., 2012) but in sensory systems activity-dependent changes to myelin are less well documented (Tagoe et al., 2014; Etxeberria et al., 2016). We investigate in mice whether axonal diameter, myelin thickness, transmitted firing rates, and conduction speed in the TB fibers are coregulated during development and whether sound-evoked activity is essential to their maturation. We provide evidence that TB axons are not mature until the fourth postnatal week and exhibit a mean g-ratio ∼0.65. As the animals approach maturity, a subpopulation of large, heavily myelinated fibers develop. Acoustic deprivation during maturation impairs the development of myelination of these fibers. Acoustic deprivation during adulthood decreases the thickness of myelin of the largest fibers, demonstrating a novel case of myelin plasticity in auditory neurons. A physiologically constrained model indicates that the thickest axons are specialized for high conduction speed, whereas the smaller axons are tuned to maintain high-frequency firing. This investigation provides further insight into the balance between the costs and benefits of thick myelination in a brain region in which the rate and timing of AP propagation is critical.

## Materials and Methods

All experimental procedures were reviewed and approved by the Bavarian district government (TVV AZ: 55.2-1-54-2532-38-13) and were performed according to the European Communities Council Directive (2010/63/EU). CBA/Ca mice were housed in a vivarium with a normal 12 h light/dark cycle. Mice of both sexes, aged postnatal day (P)8–80, were used for the physiological and anatomical experiments described below.

### 

#### 

##### *In vitro* electrophysiology.

Mice aged P8–P35 (*n* = 36) were anesthetized with isoflurane and killed by decapitation. Coronal brainstem sections (200 μm thick) containing the superior olivary complex (SOC) were cut using a Leica V1200S vibratome in ice-cold high-sucrose, low-sodium artificial CSF (ACSF). Brainstem slices were held in a slice-maintenance chamber after slicing in normal ACSF at 37°C for 30–45 min, after which they were stored at room temperature (∼22°C). Composition of the normal ACSF in mm: 125 NaCl, 2.5 KCl, 26 NaHCO_3_, 10 glucose, 1.25 NaH_2_PO_4_, 2 sodium pyruvate, 3 myo-inositol, 2 CaCl_2_, 1 MgCl_2_, and 0.5 ascorbic acid, pH 7.4 bubbled with 95% O_2_, 5% CO_2_. For the low-sodium ACSF CaCl_2_ and MgCl_2_ concentrations were 0.1 and 4 mm, respectively, and NaCl was replaced by 200 mm sucrose. Experiments were conducted at 36 ± 1°C, maintained by an inline feedback temperature controller and heated stage (TC344B, Warner Instruments) with the recording chamber being continuously perfused with ACSF at a rate of 1–2 ml/min^−1^. Whole-cell patch-clamp recordings were made from visually identified MNTB neurons (Olympus BX51WI microscope) using an EPC10/2HEKA amplifier, sampling at 50 kHz and filtering between 2.9 and 10 kHz. Patch pipettes were pulled from borosilicate glass capillaries (GC150F-7.5, OD: 1.5 mm; Harvard Apparatus) using a DMZ Universal puller (Zeitz), filled with a patch solution containing the following (in mm): 126 K-gluconate, 4 KCl, 40 HEPES, 5 EGTA, 1 MgCl_2_, 5 Na_2_ phosphocreatine, 0.2% biocytin, 292 mOsm (all chemicals from Sigma-Aldrich), pH was adjusted to 7.2 with KOH. Electrode resistance was between 2.4 and 6 MΩ. Synaptic potentials were evoked by afferent fiber stimulation with a bipolar electrode (FHC) placed at the midline over the TB fibers. For measures of conduction speed, an additional electrode was placed on the contralateral side, driven by voltage pulses generated by the HEKA amplifier and post-amplified by a linear stimulus isolator (PulseStimulator AM-2100). The second electrode was moved around the slice to achieve the lowest attainable voltage-threshold to stimulation. Latencies for the conduction speed measurements were obtained from the onset of the stimulus artifact to the half-maximum of the postsynaptic response. Only EPSCs >2 nA were considered calyceal and used for analysis. The distance between the tip of the recording electrode and the midline of the slice, and the distance between the tip of the recording electrode and the stimulation electrodes were measured using ImageJ (Schneider et al., 2012).

##### Ear-plugging procedure.

To study the influence of sensory experience on development of myelin, earplugs were inserted under MMF anesthesia at P10 (medetomidine: 0.5 mg/kg BW, midazolam: 5.0 mg/kg BW, Fentanyl: 0.05 mg/kg BW). The earplugs consisted of small pieces cut from an “EAR Classic II” human foam earplug, compressed and inserted into the external auditory meatus and sealed with dental cement (Paladur; Heraeus-Kulzer). Earplugs were examined on a daily basis and if necessary adjusted or reinserted. Earplugs were kept in place for 10 d and *in vivo* recording and auditory brainstem responses (ABRs) were obtained before and after earplug removal. Mice were then allowed to recover without earplugs for 15 or 25 d. Sham littermates received identical handling (without earplug insertion). Earplug procedure in the adult mice was performed as described for the young mice above.

##### *In vivo* measurements of neuronal activity.

Three earplug (EP)-reared and three control CBA mice (P20–P24) of both sexes were used for *in vivo* recordings. The mice were anesthetized (MMF see above). Depth of anesthesia was measured using the toe pinch reflex and animals responding were given supplemental MMF at 1/3 the initial dose. Anesthesia was maintained using this method for the duration of the experiment. The mice were then stabilized in a custom stereotaxic device. An incision was made at the top of the skull and a head post was fixed to the skull using dental cement. A craniotomy was performed above the cerebellum to access the auditory brainstem. A ground electrode was placed in the muscle at the base of the neck. Glass microelectrodes were pulled from glass capillary tubes (GC150F-7.5, Harvard Apparatus, Edenbridge, UK) so that the resistance was 5–20 MΩ when filled with 3M KCl solution or 2M potassium acetate with 2.5% biocytin. Signals were amplified (Neuroprobe Amplifier Model 1600, A-M Systems), filtered (300–3000 Hz; TDT PC1) and recorded (∼50 kHz sampling rate) with an RZ6 processor (TDT). SPIKE software (Brandon Warren, V.M. Bloedel Hearing Research Center, University of Washington) was used to calibrate the speakers (MF1, Tucker Davis Technologies), generate stimuli and record action potentials. Stimuli consisted of pure tones (50–100 ms duration, 5 ms rise/fall time) at varying intensity (0–90 dB SPL) and were presented through hollow ear bars connected to the speakers with Tygon tubing. Spike sorting and data analysis was performed offline using custom Matlab programs.

##### Auditory brainstem response recording.

For the ABR measures the anesthetized mouse (MMF see above) was placed on a heating pad powered by an ATC 1000 DC Temperature Controller (World Precision Instruments), set at 37°C in a double-walled sound-attenuated chamber (Industrial Acoustics, GmbH), which was additionally lined with acoustic foam. The loudspeaker (MF1 Tucker Davis Technologies) was calibrated with a microphone type 4938 and a preamplifier type 2670 (Brüel and Kjaer). After verification of sufficient depth of anesthesia via testing the pedal withdrawal reflex, the electrodes were inserted subdermally at the vertex (reference), over the bulla (active), and just above the hindlimb (ground). The loudspeaker was extended into the ear via a short plastic tube. Broadband clicks (0.1 ms duration, 0 ms rise/fall time) and tones of 4, 8, 16, 20, 28, 36, and 44 kHz (5 ms duration, 1 ms rise/fall time) were synthesized with Spike software (Brandon Warren, University of Washington, Seattle, WA; pre-amp gain: 20; additional gain: 0 dB) on a RZ6 Multi I/O Processor (TDT) and presented at a rate of ∼50 /s.

ABR waveforms were recorded using the RA16 PA 16 Channel Medusa preamplifier (TDT) and RZ6 Multi I/O Processor and averaged over 1000 repetitions for each frequency and intensity. Initially, a 60 dB SPL click was recorded to ensure ABRs with at least four clearly identifiable waves. Subsequently, the range of 5–55 dB SPL for clicks was tested in 5 dB steps to narrow down the possible range of thresholds, followed by 2.5 dB steps to determine the hearing threshold. Next, the frequency thresholds were established from 44 to 4 kHz using the same intensity steps given above. Thresholds for clicks and individual frequencies were established as the lowest stimulus level where any recognizable feature of the waveform was discernible, using visual inspection. Peak and trough latencies and amplitudes were analyzed using the Spike program. After completion of data collection, the mice were either woken up by subcutaneous injection of an antidote (atipamezole: 2.5 mg/kg BW; flumazenil: 0.5 mg/kg BW; naloxone: 1.2 mg/kg BW) or deeply anesthetized with Na-pentobarbital (Narcoren, Merieux: 200 mg/kg BW) and perfused transcardially for histology. Age-matched littermates were perfused as controls.

##### Axonal tracing.

Mice were anesthetized with isoflurane and killed by decapitation before the brain was removed from the skull and placed into ice-cold ACSF. TB fibers were anterogradely labeled by injecting dextran-tetramethylrhodamine (10%; MW 3000, Invitrogen) into the VCN using glass micropipettes with a tip diameter of ∼10 μm. The tracer was pressure-injected (1 bar; Picospritzer III, Parker) followed by electroporation (10 × 50 ms pulses of 50 V) using an isolated pulse stimulator (A-M Systems) to induce uptake of the dye into the bushy cells that give rise to the TB fibers including the calyces of Held. Injected brains were transferred to a chamber with oxygenated ACSF and incubated for 2.5 h at room temperature to allow for homogeneous distribution of the tracer in axons. Subsequently brains were immersion-fixed in 4% PFA for 2 h at room temperature followed overnight at 4°C before being subjected to immunocytochemistry and imaging.

##### Immunohistochemistry.

Sagittal brainstem sections including TBs of 80 μm thickness were taken from within 240 μm of the midline using a vibrating microtome (Leica, VT1200S). The tracer-injected brains were sliced coronally at 80 μm thickness. After 3 × 10 min washes in PBS, sections were transferred to a blocking solution containing 1% bovine serum albumin, 1% Triton X100, and 0.1% saponin in PBS. Tissue was incubated for 48 h at 4°C with the following primary antibodies diluted 1:100 in blocking solution: mouse monoclonal IgG1 anti-neurofilament (NF)-associated antigen, (3A10, Developmental Studies, Hybridoma Bank) and rat monoclonal IgG2a anti-myelin basic protein (MBP), (ab7349, Abcam). Coronal sections were additionally stained with antibodies directed against vesicular glutamate transporter 1 (VGLUT1; Synaptic Systems, 1:2000) to identify the calyx synapses in the MNTB. Tissue was then washed 3 × 10 min in PBS at room temperature, before incubation for 24 h at 4°C with secondary antibodies diluted 1:200 in blocking solution: Cy3 donkey anti-mouse, AlexaFluor 488 donkey anti-rat (715-545-153 and 715-166-151, respectively, Dianova) sections were rinsed 3 × 10 min in PBS, and coverslipped with Vectashield mounting medium.

##### Confocal microscopy.

Confocal optical sections were acquired with a Leica TCS SP5–2 confocal laser-scanning microscope equipped with HCX PL APO CS 20×/NA0.7 and HCX PL APO Lambda Blue 63×/NA1.4 oil-immersion objectives. For each optical section the images were collected sequentially for two fluorochromes. Stacks of 8-bit grayscale images were obtained with axial distances of 290 nm between optical sections and pixel sizes of 120–1520 nm depending on the selected zoom factor and objective. To improve the signal-to-noise ratio, images were averaged from three successive scans. The limits of resolution as calculated by lateral resolution = (0.51× wavelength of excitation)/NA (Cole et al., 2011) were 177.8 and 204.4 nm for imaging with lasers of wavelength 488 nm (NF) and 561 nm (MBP), respectively. The narrowest myelinated fibers measured in this study ≥470 nm.

##### Electron microscopy.

Three P9 mice and three P80 mice were killed with an overdose of pentobarbital and intracardially perfused with Ringer's solution. This was followed by perfusion with 2.5% glutaraldehyde plus 4% PFA in cacodylate buffer (CB). Subsequently, the brainstem was removed from the skull and postfixed in the same fixative overnight at 4°C. After washing for 3 × 10 min in CB, brainstems were sectioned parasagittally at 250 μm using a Leica V1200S vibratome. Thereafter, a 1 × 1 mm block containing the TB fibers was extracted using a razor blade. The tissue was then washed four times in CB and postfixed in 1% OsO_4_ in CB for 1–2 h. After washing and dehydrating in graded series of acetone, the tissue was embedded in Spurr's resin (Spurr, 1969). Before ultrathin (70 nm) sectioning, several semithin (1 μm) sections were cut for light microscopic investigation. The ultrathin sections were collected on Formvar-coated copper slot grids and stained with uranyl acetate and lead citrate. EM images were taken using an FEI Morgagni transmission electron microscope, (80 kV, equipped with a SIS Mega view III camera, 1375 × 1032 pixels).

##### Morphometric measures.

For the morphometric analysis of axon diameter and myelin thickness, measurements were taken using ImageJ from single confocal optical sections and also EM images of sagittal brainstem sections. Two ellipses were fit to each fiber, one to the neurofilament stained axon and one to the outside of the surrounding myelin sheath stained by myelin basic protein. The minimum diameter of the two ellipses was taken as the inner and outer axon diameters, respectively (Wang et al., 2008). The difference between inner and outer diameter (divided by two) for each individual fiber provided the thickness of the myelin sheath. To ascertain that imaging using light microscopy provided accurate measures of diameter and g-ratio, 32 pre-hearing,and 25 post-hearing axons were measured in the same way after determining by eye the edge of the axon and the edge of the outer myelin layer before fitting an ellipse.

##### Computational modeling.

We simulated a multicompartmental model of an axon consisting of 40 nodes of Ranvier and 39 interleaved internodal segments using custom-made C code. The nodes were represented by one active compartment with three active and one passive conductances, the internode was represented by two passive compartments. The active conductances in the nodes were (1) a sodium conductance modeled according to Rothman et al. (1993), (2) a high threshold potassium conductance taken from Mainen et al. (1995), and (3) a low-threshold potassium conductance (Mathews et al., 2010). All rate constants were corrected for a simulated temperature of 37°C. Parameters of the active conductances: *g*_max_ (mS/cm^2^): sodium: 588, potassium (high threshold): 60, potassium (low threshold): 60; *E*_rev_ (mV): sodium: 55, potassium −85). Geometrical and passive parameters are given in Table 1. The thickness *d* (0.0245 μm) of the myelin sheet was used to fit the speed of the axon with average geometry to the average speed.

**Table 1.** Geometrical and passive parameters of axon model

	Internode	Node
*C*^(specific)^, μF/cm^2^	0.8/ln(*r*_out_/*r*_in_)*d*/*r*_in_	0.8
*G*_leak_, mS/cm^2^	1/ln(*r*_out_/*r*_in_)*d*/*r*_in_	1
*E*_rev_, mV	−67	−67
Length, μm	198	1
Specific axial resistance, Ω/cm	100	100

Thickness of a single myelin sheet (*d*) = 0.022 μm. Internodal length and *d* were used as a fit parameters to tune conduction speed. Choice of specific axial resistance (*R*) as discussed by Lehnert et al. (2014). *r*_out_/*r*_in_, Inner and outer radius.

##### Experimental design and statistical analysis.

**Figure 1.**
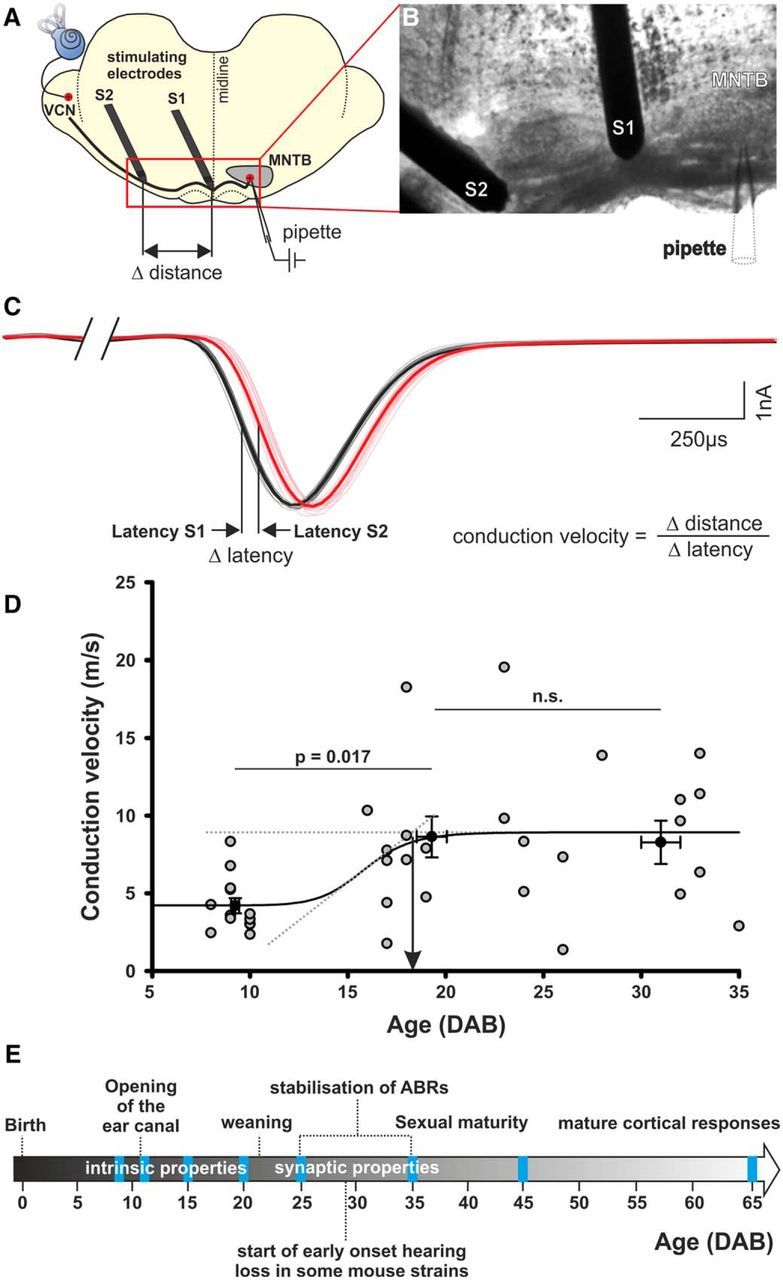
Conduction speed of TB fibers increases after hearing onset and matures ∼P18. ***A***, Schematic and (***B***) image of a coronal brainstem section showing the stimulating and recording electrodes. ***C***, Evoked EPSCs in an MNTB neuron in response to stimulation via electrode S1 (black) or S2 (red). Thicker lines are averages of 10 superimposed raw traces. Stimulating artifacts are removed for clarity. ***D***, Conduction speed of single TB fibers between P8 and P35. The dotted lines represent regression lines fitted to the slope and the plateau of the sigmoidal function. The intersection of both lines is taken as an indicator for a developmental stabilization of the conduction speed at P18.5 (arrow). ***E***, The mouse ear canal opens ∼P12 and ABRs mature by P36 (Song et al., 2006). CBA females are fertile shortly after weaning (Strong, 1936). C57 mice demonstrate loss of thresholds of single units in MNTB (Sonntag et al., 2009). In rat, response properties of auditory cortex are mature by P45 (Chang et al., 2005).

**Figure 2.**
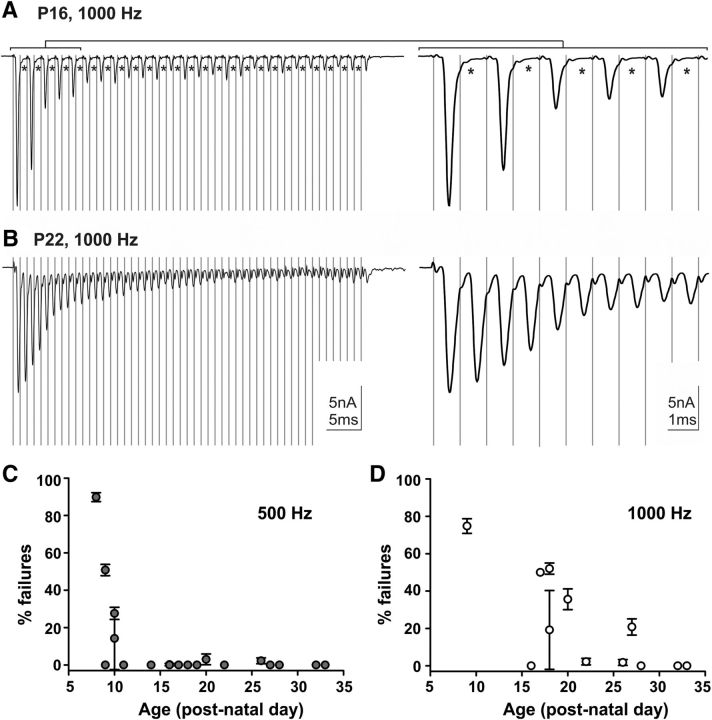
Ability of MNTB principal neurons to follow high-frequency input trains increases with postnatal age. ***A***, ***B***, Left, Example traces of calyceal EPSCs during whole-cell patch-clamp recordings from a (***A***) P16 and a (***B***) P22 neuron while stimulating TB fibers 50 times at 1000 Hz. Right, Zoom into the first 10 stimuli. Asterisks indicate failures. ***C***, ***D***, Mean percentage of failed stimulation events during 10 trains of 50 pulses at 500 Hz (***C***, circles) and 1000 Hz (***D***, triangles), plotted against postnatal age. Each symbol represents the mean percentage failures for 10 trails in a single neuron ± SEM.

**Figure 3.**
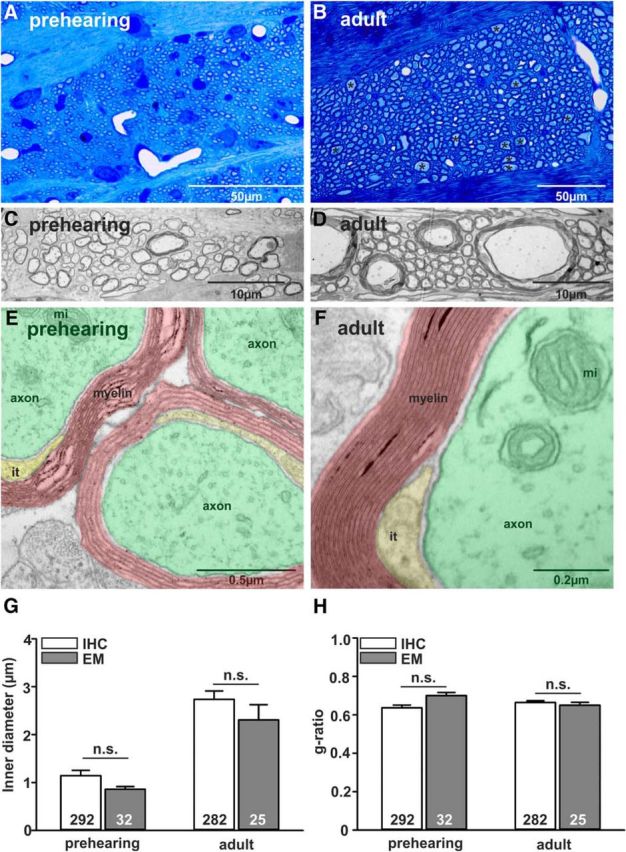
Assessment of axon diameter and g-ratio of TB fibers in prehearing (P9: ***A***, ***C***, ***E***, ***G***) and adult (P80: ***B***, ***D***, ***F***, ***H***) mice using transmission electron microscopy. ***A***, ***B***, Semithin sections are stained with Richardson's stain (methylene blue + Azur II) and provide an overview of the sagittal section through the TB. Black asterisks in ***B*** depict the location of large diameter axons. ***C***, ***D***, Magnification (22k×) shows large diameter axons in adult (***D***) but not young (***C***) mice. ***E***, ***F***, Magnification (56k×) reveals fine structures of the axons (green) like mitochondria (mi), individual layers of myelin (red), and the inner tongue of myelin (it; yellow). ***G***, ***H***, Comparison of axon diameter (***G***) and g-ratio (***H***) using either EM or immunohistochemistry revealed no significant difference for the two tested age groups.

**Figure 4.**
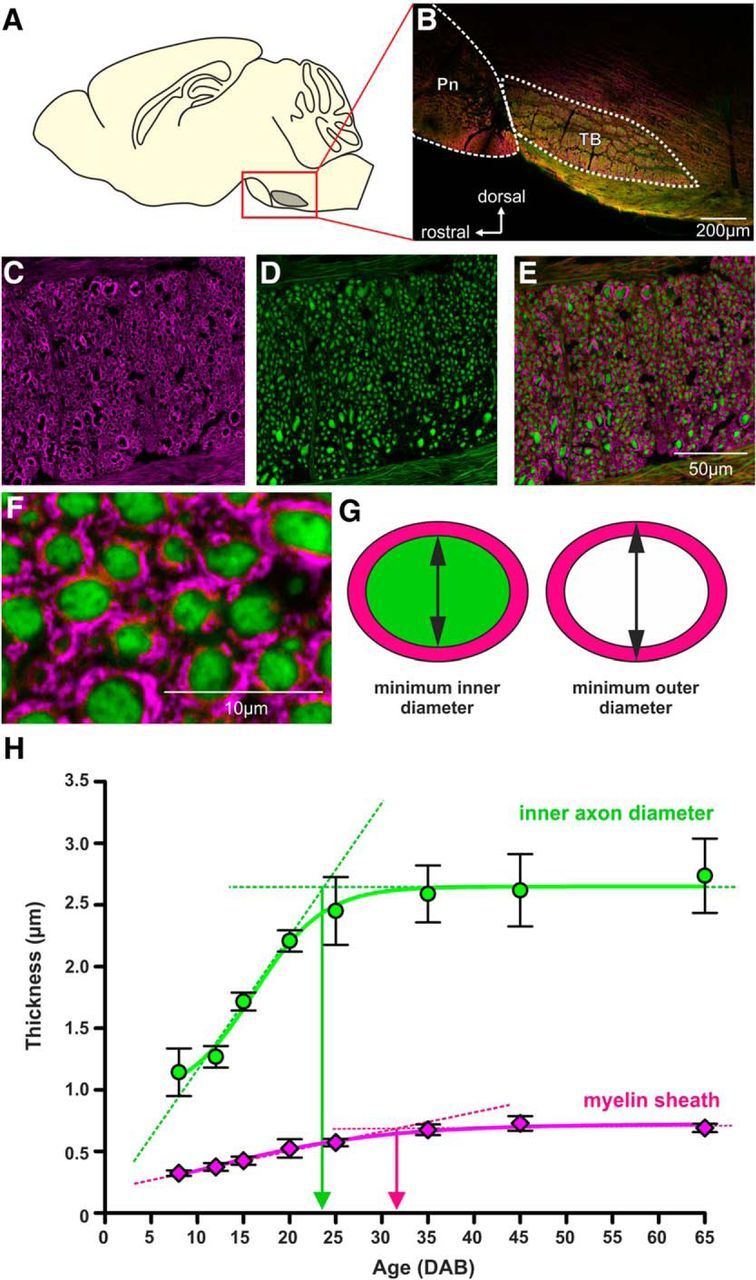
Increase of myelin sheath thickness continues beyond developmental stabilization of axon diameter. ***A***, Schematic and (***B***) image of a sagittal brainstem section at the level of the pons (Pn) and the TB. ***C***, Myelin basic protein labels the myelin sheath (magenta) around the axons. ***D***, Axons are labeled by staining against NF (green). A low-magnification overlay is shown in ***E***. ***F***, Individual axon cross sections can be identified and ellipses were fit to the inner and outer diameter of each axon (***G***). The difference between the minimum diameters for each cell was taken as a measure for the myelin sheath thickness. ***H***, Thickness of axons (green) and myelin (magenta) increases with postnatal age. The dotted lines represent linear fits to the slope and the maximum. The intercept of the two provides an approximation of the maturation age for each parameter.

**Figure 5.**
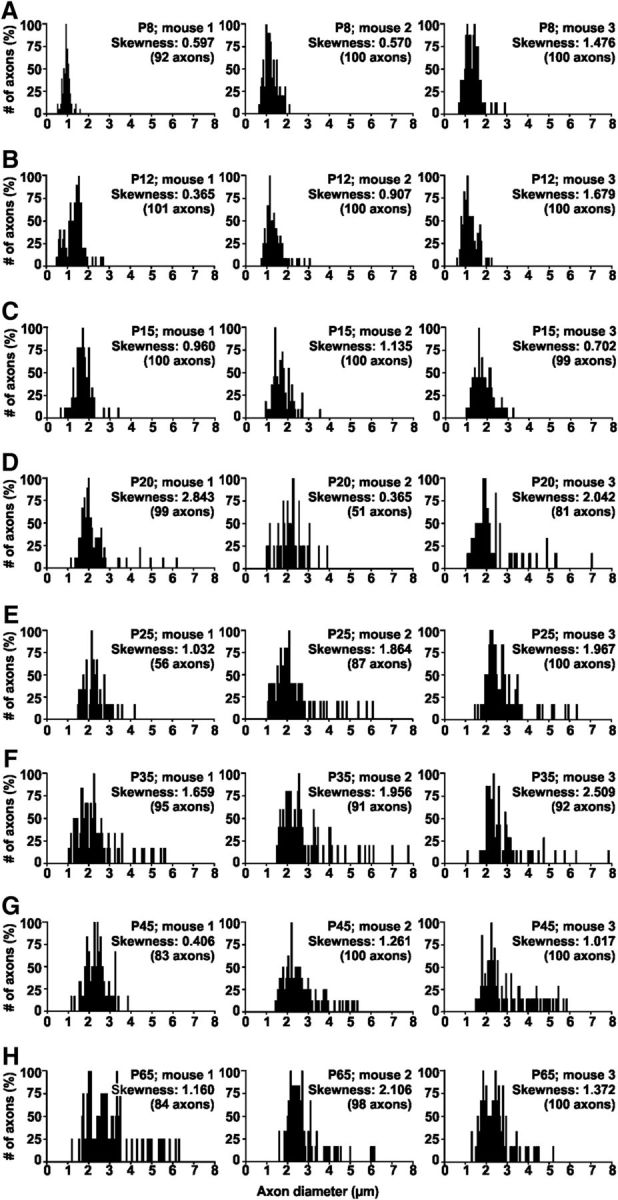
Large diameter axons first occur in the third postnatal week. Histograms show inner axon diameter for individual mice aged from P8 to P65 (***A***–***H***). A population of large diameter axons that reliably (in each animal of the age group) appears only at P20 (***D***) and older is not big enough to form a clearly double-peaked distribution. However, skewness increases with age as indicated by the skewness values in each graph.

**Figure 6.**
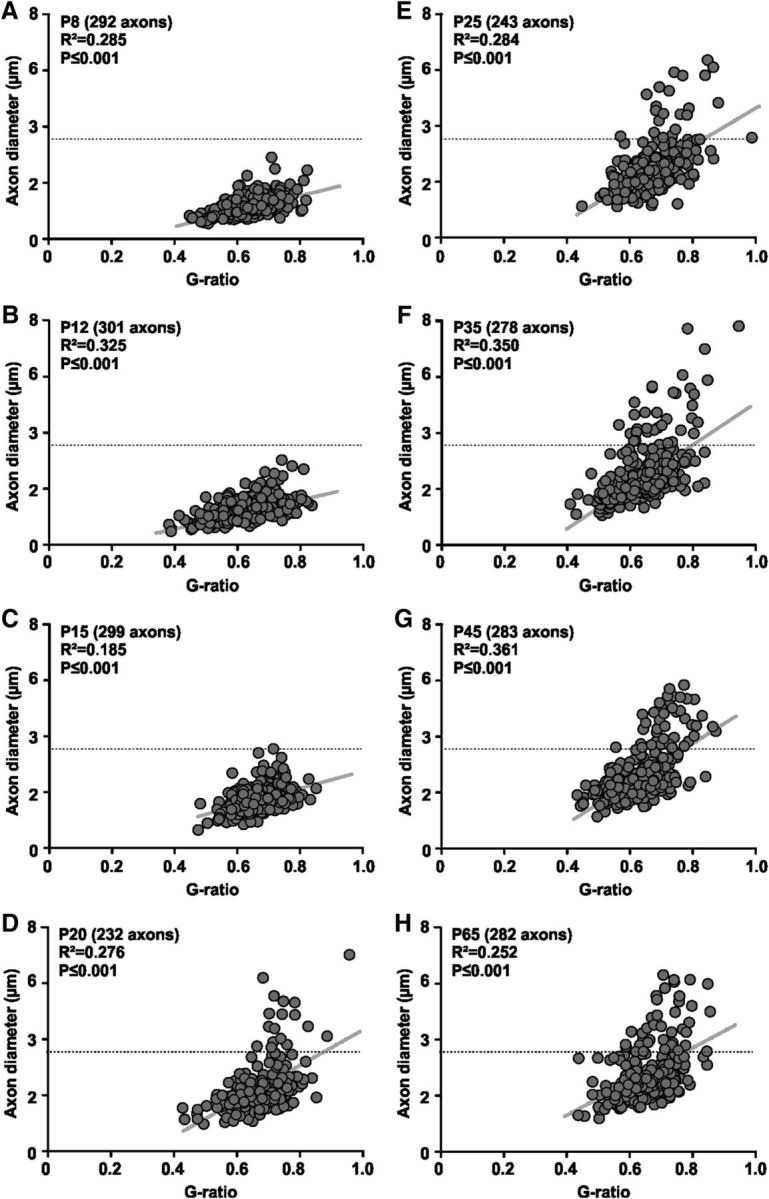
Relationship between g-ratio and axon diameter becomes strongly nonlinear for axons ≥3.5 μm. G-ratio is plotted against inner axon diameter for each individual fiber for mice aged between P8 and P65 (***A***–***H***). G-ratio and inner axon diameter show a positive correlation at all ages measured (*p* values for Pearson correlation given in the graphs). This correlation becomes progressively more nonlinear as a population of large diameter axons emerges in animals ≥P20 (***D***). For these large axons (<3.5 μm) g-ratio does not increase with diameter.

**Figure 7.**
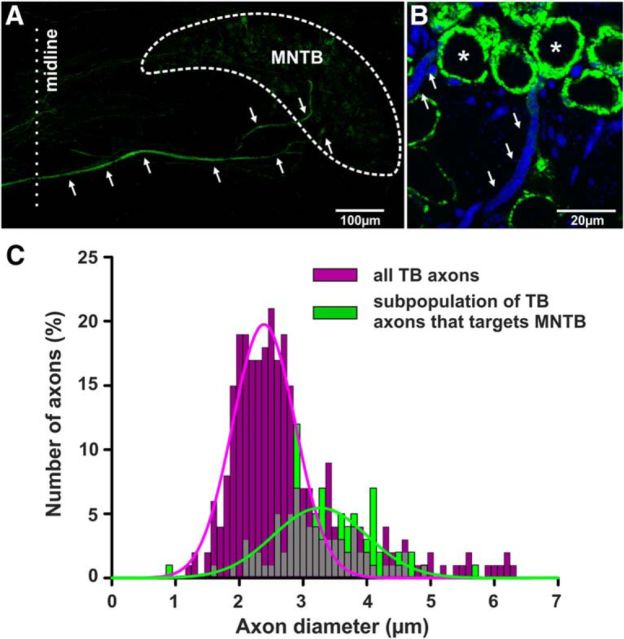
Large diameter axons primarily target MNTB neurons. ***A***, In neurotracing experiments large diameter axons could be followed from the VCN into the contralateral MNTB in coronal brainstem sections. ***B***, Large diameter axons (NF labeling in blue) giving rise to the calyx of Held (VGLUT1 labeling in green) in mouse MNTB are indicated by white arrows. ***C***, Measurements from neurotracing and immunolabeling experiments suggest that the subpopulation of large diameter axons described in Figure 5 targets MNTB neurons.

**Figure 8.**
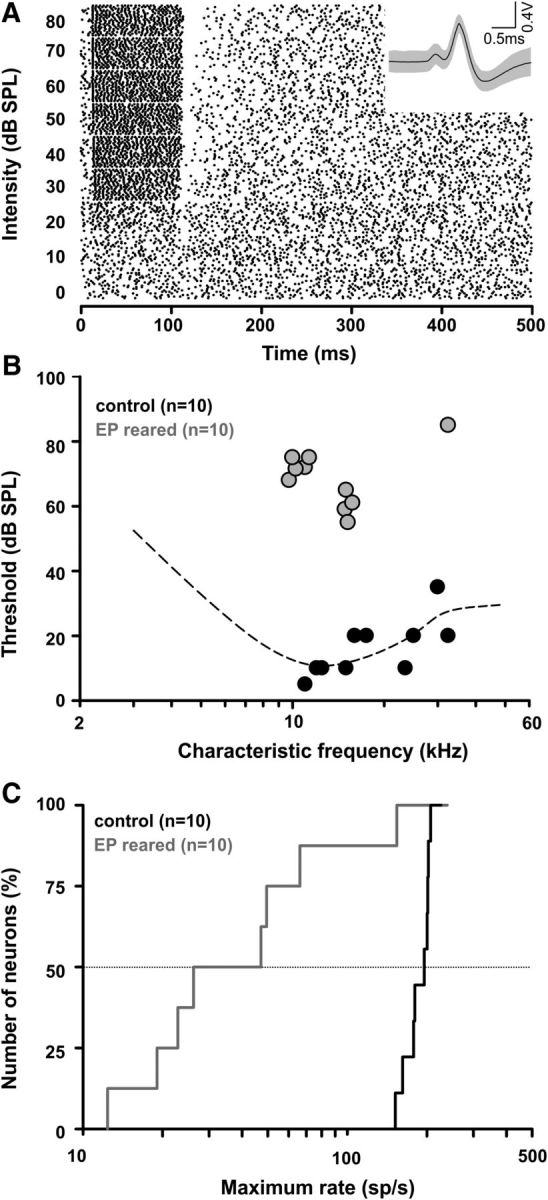
Moderate temporary sensory deprivation was induced by bilateral ear plugging. ***A***, Dot raster plot showing the spontaneous and sound-evoked responses of a single MNTB neuron of a control mouse at P20 at its characteristic frequency (15 kHz). As sound intensity increases from 0 to 80 dB SPL, at each neurons' threshold (20 dB SPL), the firing rate increases significantly beyond spontaneous firing. Inset, The typical compound waveform of MNTB recordings (Kopp-Scheinpflug et al., 2003a). ***B***, Single-unit recordings of MNTB neurons in P20–P24 mice with (gray symbols) and without (black symbols) earplugs reveal a ∼50 dB difference in auditory thresholds. Dashed line shows the behavioral audiogram of age-matched CBA control mice (Allen and Ison, 2012). ***C***, Cumulative distribution of maximum firing rates show significantly lower rates in MNTB neurons of earplugged mice.

**Figure 9.**
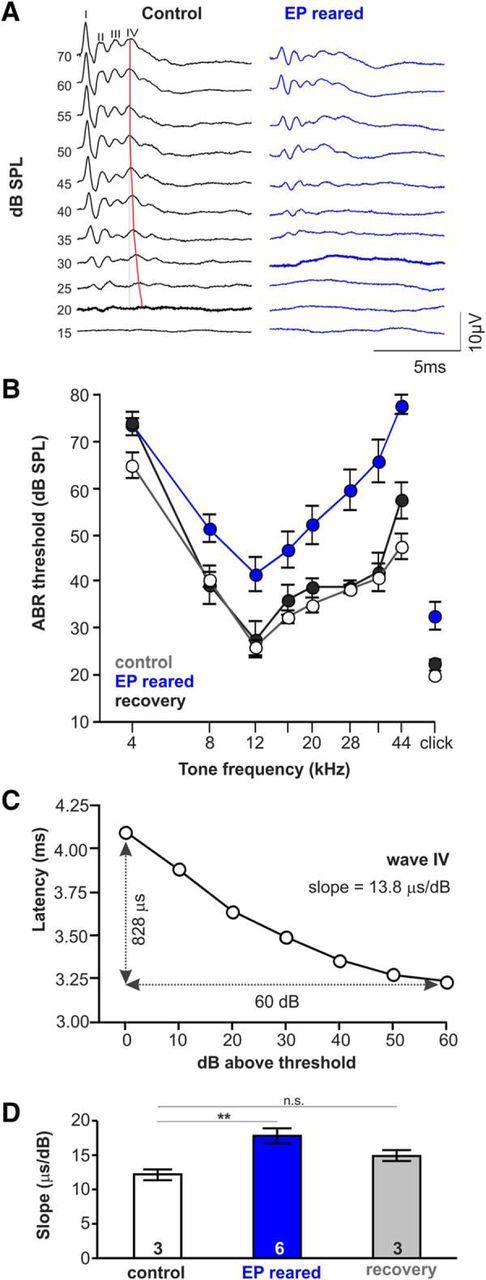
Hearing sensitivity recovers after earplug removal. ***A***, ABR recordings obtained with a click stimulus in control and EP-reared animals. Bold lines indicate threshold. ***B***, ABR thresholds of EP-reared mice were elevated after EP removal (blue). After a recovery period of 25 d (black) ABR thresholds were no longer significantly different from an non-plugged control group group (gray). ***C***, The slope of the latency-threshold curve of ABR wave IV (***A***, red line, control) can be used as an indicator for either conductive or sensorineural hearing loss (Baldwin and Watkin, 2014). ***D***, EP-reared mice had larger slopes compared with age-matched control and recovery groups.

**Figure 10.**
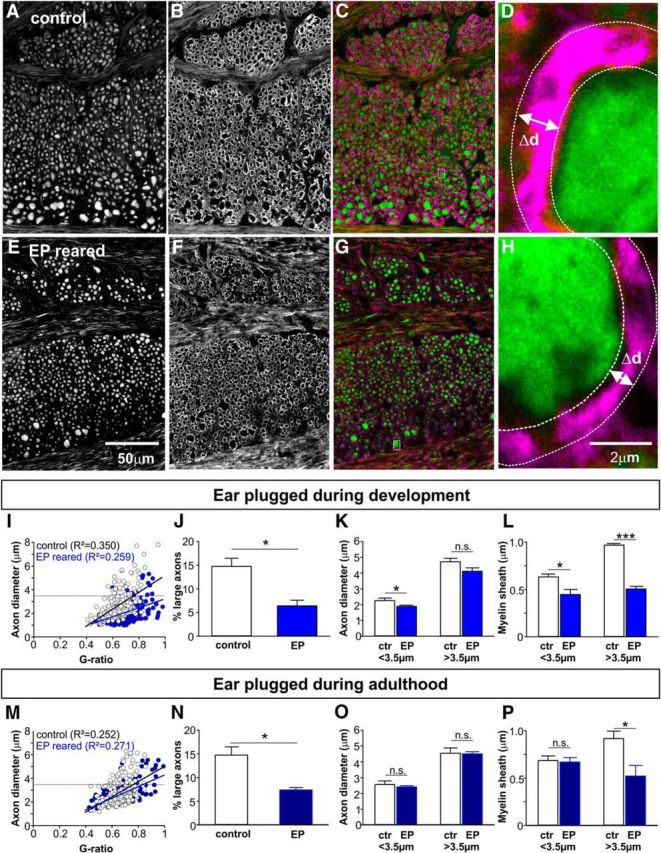
Sensory deprivation caused a reduction in the number of large diameter axons and in myelin thickness. ***A***–***D***, Control mice at P65. Sagittal section through the TB fiber tract labeled for NF (***A***) and MBP (***B***). Merge of NF (green) and MBP (magenta) shown in ***C*** low- and (***D***) high-magnification of the white rectangles in ***C***. ***E***–***H***, Mice reared with earplugs between P55–P65. Immunolabeling is the same as in ***A***–***D***. EP-reared mice have fewer large diameter axons than age-matched control mice. ***I***, G-ratio is plotted against inner axon diameter for each individual fiber for mice reared with earplugs (blue) during development (P10–P20) and age-matched controls (white). ***J***, The number of large diameter axons was reduced in EP-reared mice. ***K***, Average axon diameter was reduced in small diameter (<3.5 μm) but not large diameter (>3.5 μm) axons. ***L***, Myelin sheath thickness was reduced in both small diameter (<3.5 μm) and large diameter (>3.5 μm) axons. ***M***–***P***, Similar ear-plugging experiments in mature (P65) mice yielded comparable results: fewer large diameter axons, reduction in myelin thickness in the remaining large diameter axons, although no change in the diameter of the axons itself.

Data are presented as mean ± SEM with *p* values, degrees of freedom (df), and sample size (*n*). Statistical analyses of the data were performed with SigmaStat/SigmaPlot (SPSS Science). Comparisons between different datasets were made depending on the distribution of the data using parametric tests for normally distributed data (two-tailed Student's *t* test for comparing two groups and ANOVA for comparing three or more groups). When the normality assumption has been violated, nonparametric tests (Mann–Whitney rank sum test for comparing two groups and ANOVA on ranks for comparing three or more groups) were used. Normality was tested by the Shapiro–Wilk test. Differences were considered statistically significant at *p* < 0.05. Intrinsic properties as well as PSC amplitudes and kinetics were analyzed using Stimfit software (Guzman et al., 2014). For data acquired with patch-clamp recording (Figs. 1,2) or *in vivo* single-unit recording (see Fig. 8); *n* is the number of neurons, with one cell per brain slice (to enable anatomical reconstruction of each individual neuron), two to three brain slices per animal and at least three animals per group. For histological assessment of axon diameter and myelin thickness an average of 92 ± 2.7 axons was analyzed per animal with at least three animals per group (see Figs. 3–6, 10). Tracing and immunostaining experiments (see Fig. 7) were performed on three mice (aged P31–P36) and an average of 33.67 ± 5.78 axons were measured per animal. For data acquired with ABR recording (see Fig. 9), *n* is the number of ears tested. Again each group consisted of at least three animals. Test details are indicated in each of the respective results sections.

## Results

### Conduction speed of TB fibers increases rapidly within the first week of hearing and peaks ∼P18

A key feature of TB fibers in the mammalian brainstem is the transmission of auditory information with high firing rates. Precisely timed output of the MNTB is crucial for determination of sound source location (Grothe et al., 2010). Here we evaluate the development of conduction speed of individual TB fibers in mice aged from P8 to P35. Conduction speed was assessed by whole-cell patch-clamp recording from MNTB neurons while electrically stimulating the respective single afferent TB fiber in two locations. One stimulating electrode was placed near the midline and the other one further on the contralateral side (Fig. 1*A*,*B*). The distance between the two stimulating electrodes was divided by the difference in the latency of the resultant EPSCs when stimulating with electrode S1 or S2 (Fig. 1*A–C*; Ford et al., 2015). Before hearing onset at P12, conduction speed was 4.21 ± 0.49 m/s (age P8–P10, *n* = 13; Fig. 1*D*). Post-hearing onset, the mean conduction speed increased significantly to 8.49 ± 0.94 m/s (*n* = 24; Mann–Whitney rank sum test; *p* = 0.003). There was a difference in conduction speed between 4.21 ± 0.49 m/s (*n* = 13) at P8–P10 and 8.64 ± 1.32 (*n* = 14) at P16–P24 (df: 2, *p* = 0.017, ANOVA on ranks with Dunn's *post hoc* test). However, there was no further difference between 8.64 ± 1.32 (*n* = 14) at P16–P24 and 8.29 ± 1.67 m/s (*n* = 10) at P26–P35 (df: 2, *p* = 1, ANOVA on ranks with Dunn's *post hoc* test) indicating conduction speed to be mature ∼P18. A summary of the maturation of the auditory system is shown in Figure 1*E*.

### Ability of TB fibers to maintain high firing rates increases with postnatal age and saturates ∼P30

Though we saw no further increase in conduction speed after P18, i*n vivo* response properties and the development of the morphology of the calyx are not completely mature by this age (Ford et al., 2009; Sonntag et al., 2009). To investigate additional aspects of the development of this system, we probed the ability of the fibers to maintain high-frequency firing at increasing age. Voltage-clamp recordings were taken from postsynaptic MNTB neurons, while TB fibers, which originate from globular bushy cells in the VCN and provide calyceal (>2 nA at −60 mV) inputs (Kandler and Friauf, 1993) were subjected to pulse trains comprising 50 pulses (0.2 ms duration, at ∼2× the voltage threshold to ensure secure AP generation at 500 or 1000 Hz; Fig. 2*A*,*B*). In response to high-frequency stimulation the EPSCs showed synaptic depression and failures typical for these synapses especially in young animals (Taschenberger and von Gersdorff, 2000; Joshi and Wang, 2002). A stimulus response was considered a failure if no EPSC peak was observed in the trace during the time following one stimulus artifact and preceding the next stimulus artifact. In pre-hearing mice aged P8–P11 the TB fibers demonstrated a significantly greater failure rate than the older animals when stimulated at 500 Hz (Fig. 2*C*; failure rate_pre-hearing_ = 26.06 ± 12.78%, *n*_pre-hearing_ = 7; failure rate_post-hearing_ = 0.32 ± 0.21%, *n*_post-hearing_ = 17, Mann–Whitney rank sum test, *p* = 0.037). Of the P8–P11 group, only three of seven fibers were able to maintain 500 Hz trains without failures (10 trains, 50 pulses, 10–20 s rest between each train). The remaining four fibers suffered failure rates of at least 14% (14.2 ± 16.7%, 27.6 ± 3.2%, 89.8 ± 2.4%, 50.8 ± 3.0% failures). In contrast, 15 of 17 fibers between ages P14 and P33 showed <1% failures in response to the 500 Hz stimulation, comparable to results shown for the rat (Taschenberger and von Gersdorff, 2000). As pre-hearing fibers tended to be unable to maintain even 500 Hz trains without many failures, they were generally not subjected to 1000 Hz pulse trains. With increasing age, the fibers were progressively more able to maintain higher firing rates with fewer failures (Fig. 2*D*; Pearson correlation, *r* = 0.602, *p* = 0.0228). Three of six fibers from animals aged P16–P19 were able to support 1000 Hz firing without failure. The remaining three P16–P19 fibers suffered failures (9.6 ± 10.5, 26.0 ± 1.5, and 25.0 ± 0 failures per sweep). At P22–P33 five of six fibers were able to maintain 1000 Hz trains for 50 ms with <2% failures during the train. One P27 fiber exhibited a mean of 10.4 ± 2.2 failures per sweep. These data indicate that though conduction speed appears mature by ∼P18, the TBs' ability to maintain high-frequency firing has not yet reached an adult-like state.

Without recording presynaptically, it is impossible to determine whether the observed failure to produce EPSCs at high rates is due to failure to transmit an AP along the axon, or because of failure to effectively repolarize the presynaptic terminal or the depletion of the readily releasable pool (Wang and Kaczmarek, 1998). Though presynaptic recording is possible at the calyx of Held (Forsythe, 1994), it becomes increasingly challenging with increased age (Kim et al., 2013a). To determine whether changes in axonal characteristics contributed to the increased conduction speed and maximum firing rate, we performed a morphological study of TB axons with the aim of producing a physiologically constrained model of the mouse TB, informed by *in vitro* slice experiments and morphometry of murine TB axons.

### Developmental growth of TB axon diameter and myelin thickness continues until P25 and P35, respectively

Conduction speed and transmitted firing rates are dependent on axon diameter and myelination. Therefore we investigated the development of axon diameter and the surrounding myelin sheath of individual TB fibers in mice from P8 to P65. Generally, the method of choice to assess g-ratios (axon diameter/outer diameter of the myelinated fiber) is electron microscopy. However, because we were interested in population data of a whole fiber tract across different ages, rather than detailed, but spatially limited sections, immunohistochemistry combined with confocal imaging provided a viable alternative, allowing the study of a greater number of myelinated fibers. Thus, to validate the method, we compared g-ratio measures from sagittal sections of TB axons for two age groups (prehearing and adult) using transmission EM (Fig. 3) and immunohistochemistry/confocal imaging (Figs. 3, 4). Semithin sections were taken for an overview and better comparison with the confocal images (Fig. 3*A*,*B* vs 4*C–E*). Due to the cutting angle and the formation of the fiber tract, the axonal cross sections were not always perfectly round; therefore, for both IHC and EM images, minimum diameters were taken from ellipses fitted to the inner and outer outline of each fiber (see Materials and Methods and Fig. 4*G*). Comparing inner (axon) diameter and g-ratios assessed by using either IHC or EM revealed no significant differences within an age group (two-tailed Student's *t* test; Fig. 3*G*,*H*). Similar comparisons performed by Seidl et al. (2010) also reported no significant difference between axon diameter measures in the vertebrate CNS using light microscopy and EM.

Using IHC to investigate the development of myelin in TB fibers showed that MBP immunoreactivity could already be detected at P8. At that point it was thin and non-compact (as observed in the EM experiments; Fig. 3*E*). The EM data indicate that with maturation of the fibers myelin becomes more compact and more layers are added (Fig. 3*F*). Axon diameter and myelin thickness were measured from images of sagittal brainstem sections (Fig. 4*A*,*B*), immunostained for MBP and NF (Fig. 4*C–E*). A sigmoidal fit to the axonal diameter values and also the myelin sheath thickness values (minimum outer diameter − minimum inner diameter/2) mimicked the biological growth function of these two parameters (Fig. 4*H*). Axon diameter increased significantly from pre-hearing values of 1.14 ± 0.11 μm (292 axons, *n* = 3 mice) to a plateau of 2.45 ± 0.16 μm (243 axons, *n* = 3 mice) at P25 with no further significant changes up until P65 (Fig. 4*H*; *p* ≤ 0.001, ANOVA with Bonferroni's *post hoc* test). However, myelin thickness continues to grow until ∼P35 when an average thickness of 0.69 ± 0.01 μm (843 fibers, *n* = 9 mice, P35–P65) was reached.

### A population of large diameter TB axons develops ∼P20

Comparing the error bars for the measures taken in Figure 4*H* suggests an increase in variability of axon diameter for P20–P65 (*F*_cal_: 16.94 > *F*_crit_: 9.27; two-tailed *F* test), but this difference was not observed in the myelin sheath data (*F*_cal_: 3.72 < *F*_crit_: 9.27; two-tailed *F* test). Histograms of NF-positive axons measured in individual animals (3 per age group) were plotted (Fig. 5). As to be expected from Figure 4*H*, the center of these histograms gradually shift toward larger axon diameters (from ∼1 μm at P8 to ∼2.5 μm at P65 (ANOVA, *p* ≤ 0.001). The age where axons first grew significantly different from P8 (the youngest group tested) was P20 (multiple *t* tests with Bonferroni adjustment for multiple comparisons: *p* ≤ 0.001). At that time, a population of large diameter axons with values of up to 7.80 μm started to appear. The number of these large diameter axons was not big enough to form a clearly double peaked distribution, but was enough to cause a difference in variance (see *F* test above). These large diameter axons generated a positive skew in the histograms, with the histograms from younger age groups from P8 to P15 being significantly less skewed (skewness_P8–P15_: 0.93 ± 0.15, *n* = 9) than histograms from the older age groups (skewness_P20–P65_: 1.57 ± 0.18, *n* = 15; two-tailed Student's *t* test, *p* = 0.024, df: 22).

To characterize those large diameter axons we plotted axon diameter against g-ratio for each age group. G-ratio and axon diameter were positively correlated at all ages measured (Fig. 6*A–H*). This correlation progressively deviated from linearity as the population of large diameter axons emerged in animals older than P20 (Fig. 6*D*,*E*). For these large axons (>3.5 μm diameter) g-ratio did not increase linearly with diameter, but rather remained at ∼0.7 (Fig. 6*D–H*).

Injections of the neurotracer dextran-tetramethylrhodamine into the VCN anterogradely labeled large diameter axons that could be followed from the VCN into the contralateral MNTB in coronal brainstem sections (Fig. 7*A*), whereas smaller diameter axons that were also labeled mostly went further toward the ventral nucleus of the trapezoid body (VNTB), the medial superior olive (MSO), and the lateral superior olive (LSO). These observations are corroborated by previous studies in the TB of cats using single-fiber recordings and subsequent tracing of those into the MNTB (Brownell, 1975; Spirou et al., 1990). Axons giving rise to the calyx of Held innervating MNTB neurons were labeled with anti-NF and anti-VGLUT1 antibodies (Fig. 7*B*). Individual calyces were examined. The axons were identified by presence of VGLUT1 and NF immunoreactivity and followed away from the calyx. As the fibers were generally not perpendicular to the plane they were imaged in, the diameter of these fibers was measured as a straight line from one edge of the immunoreactive fiber to the other at what was thought to be the optical section providing the, not as the minimum diameter of an ellipse. The diameter measure was taken as far as possible away from the calyx. The mean diameter of fibers forming calyces was 3.27 ± 0.01 μm (weighted mean generated from 108 fibers, *n* = 3 mice, with 24, 34, and 50 fibers recovered from the different animals). On average, 33% of the fibers had a diameter >3.5 μm. These data show that inputs to MNTB fibers come from thicker fibers, indicating that the subpopulation of large diameter TB fibers observed in sagittal sections contribute to the population of axons innervating MNTB (Fig. 7*C*).

### Temporary sensory deprivation caused elevated auditory thresholds, reduced firing rates, and slower conduction speed

In the previous paragraphs it was demonstrated that the gain of additional acoustically driven activity that occurs at hearing onset (P12) coincides with an increase in conduction speed, axon diameter, myelin thickness, and the ability to maintain high firing rates. To better understand the function of sensory experience for structural development of the axons in the auditory pathway, in particular to test whether the observed developmental changes in axon diameter and in myelin sheath thickness were causally correlated to the onset of hearing rather than just coinciding, we then reduced the acoustic input during the period of the most prominent changes in axon diameter and myelin thickness (P10–P20). Rather than completely eliminating all sound driven activity in the auditory system, we induced a mild temporary sensory deprivation by bilateral ear plugging for a period of 10 d (from P10 to P20) which has previously been shown to affect signal processing in the auditory system (Caras and Sanes, 2015). *In vivo* single-unit recordings of MNTB neurons from EP-reared mice and non-plugged controls were assessed regarding their thresholds to auditory stimulation and their maximum firing rates at P20–P24. Spontaneous firing was assessed in the absence of acoustic stimuli. Then, the characteristic frequency (CF; i.e., the frequency of the pure tone for which the neuron has the lowest threshold) of the neuron was determined by presentation of stimuli of different frequencies and intensities. The amplitude of tones at the neurons' CF was increased stepwise until a threshold was reached where the neuron showed a significant increase in sound evoked firing compared with spontaneous activity (Fig. 8*A*). With further increasing intensity, firing rates also increased. The typical compound waveform of MNTB single-unit recordings (Fig. 8*A*, inset) comprised the monophasic presynaptic action potential followed by the synaptic delay and the biphasic postsynaptic action potential (Kopp-Scheinpflug et al., 2003a) and was taken as a confirmation of recording from MNTB neurons. Recordings of MNTB neurons at P20–P24 from control mice closely match the behavioral audiogram of age-matched mice (Allen and Ison, 2012). Auditory thresholds of MNTB neurons in EP-reared mice were significantly elevated by 52.5 dB from an average of 17.9 ± 2.3 dB SPL (*n* = 12) in controls to 70.5 ± 3.2 dB SPL (*n* = 11) in earplugged mice (two-tailed Student's *t* test, *p* < 0.001, df: 21; Fig. 8*B*). Maximum firing rates transmitted along TB fibers were also assessed by recording from MNTB neurons with a clear pre-potential. EP-reared mice had significantly lower (70.7 ± 25.4 spikes/s, *n* = 9) maximum firing rates than non-plugged littermates (177.7 ± 9.3 spikes/s, *n* = 12; two-tailed Student's *t* test: *p* < 0.001, df: 19; Fig. 8*C*), corroborating a decrease in activity following earplugging rather than an increase in firing activity due to possible compensatory mechanisms (Barclay et al., 2016; Clarkson et al., 2016).

Earplugging was chosen as means of temporary sensory deprivation over deafening to avoid effects of degeneration of axons and neurons following hair-cell death. Reversibility of the earplugging procedure was evaluated by ABR recordings. Animals raised with earplugs had significantly elevated ABR thresholds at P35 (Fig. 9*A*,*B*) which recovered back to levels of non-plugged controls by P45 (Fig. 9*A*,*B*). ABR recordings also allow estimation of conduction speed through the auditory brainstem (Kim et al., 2013b). However, as latency changes with intensity and the observed changes in threshold would affect latency, it was important to assess slopes of the latency-intensity functions rather than absolute latencies (Fig. 9*A*). The latencies of ABR peaks IV of control mice plotted as a function of decibels above threshold creates a curve whose slope (Fig. 9*C*) is used as a measure of hearing loss in human audiology (Baldwin and Watkin, 2014): a parallel shift in the latency-threshold curve of ABR wave IV is suggested to correspond to conductive hearing loss, while an increase in slope indicates sensorineural hearing loss (Fig. 9*D*). In control mice (*n* = 3) the slope generated by the decreasing latency of wave IV, (calculated as the mean of the slope generated at each frequency tested, as in Fig. 9*B*, for both ears of each mouse) was significantly lower than those recorded at P35 from mice raised with ear plugs (*n* = 6), but not significantly different from that of mice raised with ear plugs that were allowed to recover for a further 10 d (*n* = 3; control: 12.2 ± 0.8 μs/dB; plugged: 17.8 ± 1.1 μs/dB; recovery: 15.0 ± 0.8 μs/dB; df: 2, *p* ≤ 0.001; ANOVA on ranks with Dunn's *post hoc* test). Our data show that temporary sensory deprivation resulted in a larger slope suggesting sensorineuronal hearing loss.

### Temporary sensory deprivation resulted in fewer large diameter axons and thinner myelin while maintaining axon diameter

Large diameter axons are located ventrally in the TB (Fig. 10*A*) and are known to target MNTB neurons (Brownell, 1975; Spirou et al., 1990). When comparing axon diameter and myelin thickness of TB fibers from EP-reared (Fig. 10*A–D*) and control animals (Fig. 10*E–H*), the number of large diameter axons was reduced (Fig. 10*A*,*E*). The population data show a significant reduction in the number of axons >3.5 μm diameter for mice that were earplugged during development between P10 and P20 from 13.3 ± 1.6% in control mice (*n* = 3) to 6.4 ± 1.0% in EP-reared mice (*n* = 3, *p* = 0.022, df: 4, two-tailed Student's *t* test; Fig. 10*I*,*J*). Analyzing changes in axon diameter and myelin thickness separately for small (<3.5 μm diameter) and large (>3.5 μm diameter) fibers revealed a slight decrease in axon diameter for the small axons (control: 2.3 ± 0.11 μm, *n* = 3; EP-reared: 1.89 ± 0.01 μm; *n* = 4, *p* = 0.013, df: 5, two-tailed Student's *t* test), but no change in diameter for the large axons (control: 4.72 ± 0.18 μm, *n* = 3; EP-reared: 4.2 ± 0.21 μm; *n* = 3, df: 4, *p* = 0.099, two-tailed Student's *t* test; Fig. 10*K*). The thickness of the myelin sheath is, however, significantly reduced in both groups of axons suggesting that the development of myelin in the TB depends on sound-driven activity (<3.5 μm: *p* = 0.037, df: 5, two-tailed Student's *t* test; >3.5 μm: *p* ≤ 0.001, df: 4, two-tailed Student's *t* test; Fig. 10*L*).

Repeating a similar ear-plugging procedure in adult mice, again resulted in a decrease in the number of large diameter axons from 14.7 ± 1.7% in controls (*n* = 3) to 7.7 ± 0.3% (*n* = 3) in earplugged mice (*p* = 0.014, df: 5, two-tailed Student's *t* test; Fig. 10*E*,*M*,*N*). The large diameter axons which remained to have significantly thinner myelin (0.52 ± 0.11 μm; *n* = 3) than age-matched controls (0.92 ± 0.07 μm; *n* = 3, df: 4, *p* = 0.040, two-tailed Student's *t* test; Fig. 10*P*). Axon diameter for these axons remained unchanged (control: 4.54 ± 0.32 μm, *n* = 3; EP-reared: 4.49 ± 0.12 μm, *n* = 3; *p* = 0.898, df: 4, two-tailed Student's *t* test; Fig. 10 *O*).

### Modeling suggests an impact of sensory deprivation on both the ability to maintain high firing rates and on conduction speed

**Figure 11.**
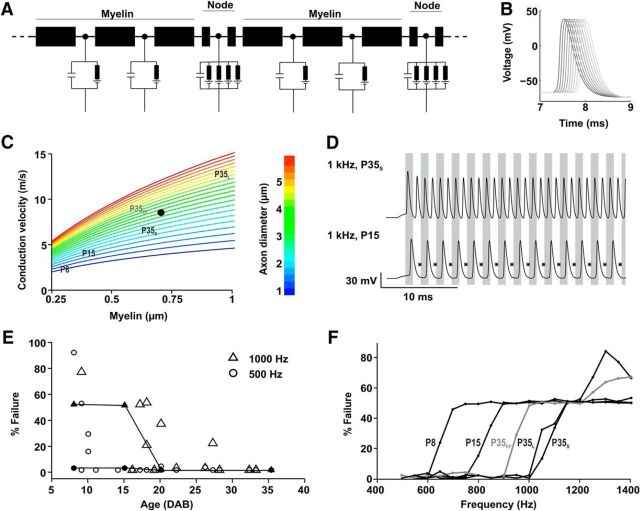
Effects of Myelin thickness and axon diameter in a computational model. ***A***, The multicompartmental model consists of 40 nodes of Ranvier (only 2 shown) with active conductances. The myelinated part between the nodes are each separated into two passive compartments. ***B***, Example voltage traces during an AP. Shown are traces from every third node of Ranvier from node 6 (black) to 33 (gray). ***C***, Conduction speed as a function of myelin thickness and axon diameter (colors). The black dot indicates the average axon geometry of MNTB innervating fibers (inner 3.25, outer 4.65, g-ratio 0.70), which is used to fit the average conduction speed (8.8 m/s; Fig. 1). The labels indicate five different experimental conditions: P8 (inner: 1.1 μm, outer: 1.7 μm, g-ratio: 0.65); P15 (inner: 1.7 μm, outer: 2.5 μm, g-ratio: 0.68); P35_S_ small/thin diameter axons (inner: 2.3 μm, outer: 3.6 μm, g-ratio: 0.64); P35_L_ large/thick diameter axons (inner: 4.7 μm, outer: 6.6 μm, g-ratio: 0.71); P35_EP_ thick axons, earplugged (inner: 4.2 μm, outer: 5.4 μm, g-ratio: 0.78). Inset, Graphical inset of fiber geometries. ***D***, Two example voltage traces obtained from stimulation of the first node of Ranvier with a 1000 Hz train of current pulses at an amplitude of twice the rheobase. Asterisks indicate skipped cycles for P16 mice. Gray and white vertical bars mark stimulation period. ***E***, Measured and predicted failures as a function of age. Open symbols are measured data (as in Fig. 2). Filled symbols and lines are model predictions assuming the average axon geometry in the age groups P8, P15, P20, and P35. ***F***, Predicted failures as a function of stimulus frequency for the average values measured in five experimental conditions (as in ***C***).

The occurrence of a population of axons that falls outside the linear relationship between axon diameter and g-ratio raises questions about the balance between spatial constraints, metabolic costs, and limitations to speed and precision in conducting trains of stimuli. We therefore developed a multicompartmental, physiologically constrained model of the axon (Fig. 11*A*,*B*) to test whether the observed developmental and activity-dependent changes in axon thickness and g-ratio had an appreciable impact on axonal conduction and transmitted rates. A range of axon diameters, between 1 and 6 μm were simulated. Figure 11*C* displays the predicted conduction speed for a range of different axons diameters. The model predicts that for the thickest axons, having a thicker myelin sheath would increase conduction speed by >7 m/s. Potential increases in conduction speed for thinner axons were smaller: for the thinnest axons, even having 1 μm of myelin would increase conduction speed by <2 m/s. Each annotation represents the mean axon diameter from a different age group or condition based on the measurements taken from the immunolabeled tissue. The annotations are arranged at a position on the *x*-axis corresponding to the mean thickness of myelin at this developmental stage: mean axon diameter at P8 (inner: 1.1 μm, myelin: 0.3 μm, predicted speed: 2.8 m/s), mean axon diameter at P15 (inner: 1.7 μm, myelin: 0.35, predicted speed: 4.8 m/s), P35_S_: mean axon diameter at P35 calculated from the population <3.5 μm (2.3 μm, myelin: 0.65 μm, predicted speed: 7.4 m/s), P35_L_: mean diameter of axons >3.5 μm following normal development (no ear plugging: 4.7 μm, myelin: 0.85 μm, predicted speed: 14.5 m/s), P35_EP_: mean diameter of axons <3.5 μm following ear plugging (4.2 μm, myelin: 0.6, predicted speed: 10.4 m/s).

TB axons are able to maintain high-frequency AP trains. We tested how the model responds to high-frequency AP trains to probe the relationships between diameter, g-ratio, and failure rate. Figure 11*E* shows *in vitro* data from Figure 2 to allow comparison to the model. The lines indicate the model's performance with average geometrical parameters from P8, P15, P25, and P35 during 1000 Hz (triangles) and 500 Hz (circles) AP trains, respectively. The model was also tested with AP trains from 500 to 1500 Hz (at 50 Hz intervals, e.g., 500, 550, 600 Hz, etc.) to predict at which frequency the different classes of axon fail to follow the stimulus effectively. The axons based on P8 fibers, being the smallest and least myelinated, supported lowest firing rates. Generally lower g-ratio (more myelin) leads to increased maximum rate of firing. Increasing the axon diameter without adding myelin, however, reduces firing rate as the axial leak at the nodes of Ranvier increases the effective charging time and thus induces a higher percentage of failures. Therefore P35_S_ axons <3.5 μm might be even slightly better suited to follow high-frequency firing than the thicker axons (>3.5 μm) also observed at P35_L_ (Fig. 11*F*). To summarize, the data show the growth in axon diameter between P8 and P18 is more crucial for speed increase, while the following thickening of myelin increases maximum firing rates. As the model shows a good prediction of conduction speed and firing rates along TB fibers for the different developmental stages, in the next step it was used to assess the impact of sensory deprivation on these two parameters. The model suggests that in the mature system (P35) sensory deprivation would cause an ∼30% decrease in conduction speed (Fig. 11*C*), whereas the ability to maintain high-frequency firing ends at a 10% lower frequency (Fig. 11*F*).

## Discussion

The present study characterized the postnatal development of TB fibers based on their conduction speed, transmitted firing rates, inner axon diameter, and myelin thickness and studied the impact of temporary sensory deprivation, to better understand the role of sensory experience on structure–function relationships of axons in mammalian sensory systems. From these data, it is possible to draw the subsequent conclusions about the series of events in axonal maturation during increased TB activity following hearing onset at P12 (Sonntag et al., 2009). (1) Conduction speed is stable by ∼P18. (2) Axon diameter increases across hearing onset and is stable by ∼P25. (3) The ability to follow high-frequency firing and myelin thickness increase across hearing onset and reach a plateau by ∼P35. (4) Temporary sensory deprivation beginning at P10 reduces activity and prevents normal developmental thickening of axon and myelin. Sensory deprivation also reduces myelin in adults.

Auditory brainstem fibers are known for their fast and temporally precise transmission, and high firing rates (Carr and Soares, 2002; Perge et al., 2009, 2012; Seidl et al., 2010; Kim et al., 2013a; Ford et al., 2015; Seidl and Rubel, 2016). Though conduction velocities of up to 100 m/s have been reported for myelinated fibers with diameters of at least 10 μm (Firmin et al., 2014); the 8.49 m/s found here for TB fibers is consistent with observations in auditory brainstem axons of other small animal models, such as chickens (Seidl et al., 2010), owls (Carr and Konishi, 1990), and gerbils (Ford et al., 2015). Given an estimate of 4.5 mm from the VCN in the mouse to the contralateral MNTB, a conduction speed of 9 m/s will transmit an action potential from VCN to MNTB in ∼0.5 ms. This value is very close to the difference in acoustically evoked action potential latencies between MNTB (4.2 ± 0.6 ms) and VCN (3.8 ± 0.2 ms) of mice *in vivo* (Kopp-Scheinpflug et al., 2003b) and thus match the requirements of the system.

### Nonlinearity of largest fibers

Ford et al. (2015) reported TB fibers in gerbil with diameters between 1.7 and 5.5 μm, which are comparable to the measurements of the present study. G-ratio values of the present study are also in line with previously published values for CNS of ∼0.6–0.8 (Chatzopoulou et al., 2008; Ford et al., 2015; Stikov et al., 2015; Seidl and Rubel, 2016). Rushton (1951) predicted that a g-ratio of 0.6 was optimal for maximizing conduction speed. Waxman and Bennett (1972) questioned its validity in CNS, and argued that maximization of conduction speed is not the only criterion. One unexpected result in our data was the loss of the linear relationship between diameter and g-ratio for the large diameter fibers. A breakdown of the linear diameter-to-g-ratio relationship has been previously reported (Hildebrand and Hahn, 1978; Stikov et al., 2015) for large fibers but the functional consequence of this alteration was not shown. The large amount of myelin in the large diameter axons and the observation that acoustic deprivation causes decreased myelin thickness lends support to the idea that thick myelin on large axons is required to sustain high firing rates at fast speed, but is metabolically expensive to construct and support. Therefore it is only sustained when fibers need to fire at high frequencies to make the tradeoff between the costs and benefits of the thick myelin profitable (Harris and Attwell, 2012). The earplugging in the present study caused an average auditory threshold elevation of 50 dB. Because intensity is encoded in firing rate, lower intensities due to the earplugs resulted in decreased firing rates in auditory brainstem neurons (Fig. 8). The resultant lower firing rates would require less myelination to still ensure sufficient speed and a low failure rate, but how is the reduction in myelin achieved?

### Possible mechanisms for activity-dependent increase or decrease of myelin

Activity-dependent myelin plasticity has been reported in different systems and studies generally agree that release of glutamate or ATP from electrically active axons causes depolarization of oligodendrocytes, their subsequent differentiation and finally increased myelination of active axons (Fields and Ni, 2010; Wake et al., 2011; Gibson et al., 2014). However, little is known about how myelin is removed from less active axons. Reports of thinner myelin as a result of less activity are often intermingled with a lack or delay of normal developmental thickening of myelin. Mice deprived of social contact during the 2 weeks postweaning exhibited thinner myelination for axons of the prefrontal cortex along with cognitive and behavioral deficits (Makinodan et al., 2012), whereas social deprivation in adult animals affected myelin but did not result in behavioral changes (Liu et al., 2012). In the developing visual system, monocular deprivation or genetic attenuation of VGLUT2 prevents development of normal internodal length in fibers of the optic nerve, with no change in myelin thickness (Etxeberria et al., 2016). Correspondingly, blocking the ears could alter glutamatergic transmission (Barclay et al., 2016; Clarkson et al., 2016) and prevent the developmental process of glutamate-driven upregulation of oligodendrocyte activity resulting in thinner myelin. Several signaling pathways have been implicated in activity-dependent upregulation of myelin during development, for example Ishii et al., (2012) demonstrate that pathways driven by the extracellular signal-regulated protein kinases, ERK1 and ERK2, are responsible for an increase in the thickness of myelin sheaths in mouse CNS. Activation of ERK1/2 increases myelin thickness, conduction speed, and hippocampal-dependent acquisition of memory in adult mice (Jeffries et al., 2016), so vice versa blocking ERK1/2 kinases might be a good direction to study the mechanisms that cause reduction of myelin.

Prevention of processes normally driven by development will impact the cohort of mice in the present study that were reared with earplugs during development but does not explain the reduction in myelin of the large diameter axons following ear plugging in adult mice. Demyelination has been demonstrated in the auditory nerve following traumatic exposure to sound (Tagoe et al., 2014), however, here earplugging was selected specifically because it was not traumatic. The decreased thickness of myelin in response to earplugging adult mice may represent an atrophy of myelin due to inactivity, a phenomena that has been reported for peripheral soleus nerves in adult rat (Canu et al., 2009) but is poorly documented in the CNS. A recent study suggests that the blockade of GABAergic transmission contributes to demyelination but the exact mechanism is still elusive (Hamilton et al., 2017).

The reduction of sound-evoked activity not only reduced the thickness of the myelin sheath, it also lowered the number of large diameter axons. Loss of peripheral sensitivity induces changes in the central auditory system (Willott et al., 1991; O'Neill et al., 1997; Zettel et al., 2007; Werthat et al., 2008). Loss of Schwann cells in the peripheral nervous system causes decreased neurofilament density, phosphorylation, and axon diameter (Cole et al., 1994). It is possible that the thick axons of TB fibers are maintained by a high activity state, either by mechanisms intrinsic to the neuron, or via activity-dependent interaction with the myelinating oligodendrocytes.

### What would be the consequences if loss of myelination was a common feature following elevation of peripheral thresholds?

The inner axon diameter, the myelin thickness, and the distance between the nodes of Ranvier determine the conduction speed and are highly specialized in auditory fibers (Seidl et al., 2010; Kim et al., 2013a; Ford et al., 2015; Seidl and Rubel, 2016). Adaptations in the axon diameter and internodal distance according to their site of termination within the MNTB as they were described for especially fast conducting, low-frequency TB axons of the gerbil (Ford et al., 2015), were not found in mice which lack this specialized low-frequency hearing (Stange-Marten et al., 2017). Thus, as myelin patterning and conduction speed in mouse TB fibers seem independent of the axonal tonotopic termination site (Stange-Marten et al., 2017), in the present study axons were not differentiated based on their medial versus lateral termination site. Conducting a comparable study as presented here for the mouse, in the gerbil would be useful in determining whether the adaptations observed in this study are specific to high- or low-frequency hearing. The above specializations emphasize once more that adaptations in myelination do not necessarily mean maximizing the values for myelin thickness or conduction speed. It rather is an optimization to achieve the conduction speed and transmitted firing rates that are necessary to perform auditory function while staying within the limits of space and metabolic demand (Perge et al., 2009, 2012). The need for tuning of conduction speed is great in auditory brainstem nuclei due to the nature of the tasks they perform: precisely timed inhibition from the MNTB is necessary for encoding gaps in sound and detection of interaural timing and intensity differences. If loss of myelin following peripheral compromise is a common feature across species, it could contribute significantly to explanation of problems that accompany loss of absolute thresholds in humans, such as difficulty understanding speech in noise.

### Future directions

An informative next step in investigating mechanisms underlying myelin reduction in mature animals would be extending the study to EM level of myelin fine architecture to determine whether a difference in number or thickness of individual lamella accounts for the reduction in myelin following sensory deprivation. Additionally, comparing TB fibers from developing animals when myelin is still increasing with those of mature animals would be useful to determine whether vesicles containing glutamate, ATP, or GABA are present in the axons and if these could account for some of the axo-glial signaling during development and sensory deprivation. With a wider view, it would be valuable to examine myelin changes following age-related hearing loss, or in cochlea implant users to determine whether these conditions result in failure to maintain myelin in downstream CNS structures.
